# Phenotypic and genetic characterization of daptomycin non-susceptible *Staphylococcus aureus* strains selected by adaptive laboratory evolution

**DOI:** 10.3389/fcimb.2024.1453233

**Published:** 2024-10-24

**Authors:** Yanlei Xu, Yanghua Xiao, Huilin Zhao, Bingjie Wang, Jingyi Yu, Yongpeng Shang, Ying Zhou, Xiaocui Wu, Yinjuan Guo, Fangyou Yu

**Affiliations:** Department of Clinical Laboratory Medicine, Shanghai Pulmonary Hospital, School of Medicine, Tongji University, Shanghai, China

**Keywords:** *Staphylococcus aureus*, daptomycin, non-susceptible, *mprF*, *saeR*

## Abstract

**Background:**

Daptomycin non-susceptible *Staphylococcus aureus* (DNS) strains pose a serious clinical threat, yet their characteristics remain poorly understood.

**Methods:**

DNS derivatives were generated by exposing *S. aureus* strains to subinhibitory concentrations of daptomycin. Competition experiment and growth kinetics experiment were used to observe the growth of bacteria. *Galleria mellonella* larvae and mouse skin abscess models were used to observe the virulence of bacteria. Transmission electron microscopy (TEM), cytochrome C experiment and biofilm formation experiment were used to observe the drug resistance phenotype. And homologous recombination was used to study the role of mutations.

**Results:**

Phenotypic profiling of DNS strains revealed impaired growth, increased cell wall thickness, enhanced biofilm formation, reduced negative surface charge, and attenuated virulence compared to their wild-type strains. Whole genome sequencing identified mutations in *mprF*, *cls2*, and *saeR* in DNS strains. Allelic replacement experiments validated the roles of MprF L341F and Cls2 F60S substitutions in augmenting daptomycin non-susceptibility in Newman. Deletion of *saeR* in the Newman_MprFL341F_ strain and complementation of *saeR* in the Newman-DNS strain did not directly alter daptomycin susceptibility. However, the deletion of *saeR* was found to enhance competitive fitness under daptomycin pressure.

**Conclusion:**

This work validates adaptive laboratory evolution (ALE) for modeling clinical DNS strains and uncovers contributions of *mprF*, *cls2*, and *saeR* mutations to the adaptation and resistance mechanisms of *S. aureus* against daptomycin. These findings enrich our understanding of how *S. aureus* acquired resistance to daptomycin, thus paving the way for the development of more effective treatment strategies and offering potential molecular markers for resistance surveillance.

## Introduction

Daptomycin, a cyclic lipopeptide antibiotic, is a key player in battling against gram-positive pathogens. Initially, daptomycin non-susceptible *Staphylococcus aureus* (DNS) strains were rare in clinical practice. However, recent reports have highlighted an upswing in the emergence of these strains during treatment (Sharma et al., 2008; Parra-Ruiz et al., 2010).

The mechanisms of DNS strains are yet to be completely understood, but described phenotypic changes include thickened cell walls, enhanced positive cell surface charge due to altered membrane lipid composition (Yang et al., 2010; Mishra et al., 2014; Ma et al., 2018). At the molecular level, mutations in genes involved in membrane phospholipid metabolism, RNA polymerase subunits and cell wall synthesis can give rise to daptomycin resistance (Howden et al., 2011; Peleg et al., 2012; Su et al., 2015). Among these, mutations in genes associated with membrane phospholipid metabolism (e.g., *mprF*, *cls2*) were the most common in clinical DNS strains. MprF (multiple peptide resistance factor) catalyzes the conversion of phosphatidyl glycerol (PG) into lysine-phosphatidyl glycerol (L-PG) (Ernst and Peschel, 2011). Cls2 is one of two cardiolipin synthases in *S. aureus* that catalyzes the synthesis of cardiolipin by PG (Short and White, 1972). Mutations in the *cls2* gene can significantly impact lipid composition and bacterial membrane properties (Jiang et al., 2019). The reduction of PG content leads to a decrease in the negative charge of the bacterial surface, allowing repulsion of positively charged daptomycin-Ca^2+^ complex and promoting resistance (Julian et al., 2010; Peleg et al., 2012). The SaeRS two-component signal transduction system plays a crucial role in regulating various virulence factor expression in *S. aureus* during infection (Liang et al., 2006; Miller et al., 2021). While previous studies have identified *saeR* mutations in DNS strains, the exact role of *saeR* in conferring daptomycin non-susceptibility has yet to be fully elucidated.

Adaptive Laboratory Evolution (ALE) is an experimental technique employed to iteratively cultivate microorganisms, driving their gradual evolution in specific environments to acquire desired traits (Peschel et al., 1999; Dorau et al., 2021; Mavrommati et al., 2022). This method is significant for studying the evolution and adaptability of microorganisms under different conditions, providing valuable insights into their genetic and phenotypic changes.

In this study, we employ the ALE strategy to construct three DNS strains and characterize their phenotypic and genomic characteristics. In our study, we identified mutations in *mprF*, *cls2*, and *saeR* in the DNS strains. We further dissected the individual impacts of these mutations on daptomycin susceptibility using homologous recombination, providing valuable insights into the machinery of antibiotic resistance.

## Materials and methods

### Bacterial strains and antibiotic

*S.aureus* strains USA300 (LAC), Newman and SA75 were used as WT strains to generate DNS strains (Juliane et al., 2007; Tadashi et al., 2007). *S. aureus* strain SA75 was isolated from a patient with a purulent skin infection at the First Affiliated Hospital of Wenzhou Medical University. Daptomycin was purchase from MedChemExpress. DNS strains were selected *in vitro* via ALE by continuously culturing WT strains in sub-inhibitory concentrations of daptomycin. The specific method is as follows: Firstly, ½ the minimum inhibitory concentration (MIC) of daptomycin was used to induce bacterial resistance to daptomycin. The induction conditions were, the strains were incubated at 37°C with shaking at 220 rpm for 24 h in Mueller-Hinton broth (MHB; BD, USA) which contain ½ the MIC of daptomycin and 50 mg/L of calcium. After each 5 generations we determined the bacterial MIC of daptomycin. On the day the MIC was determined, we performed induction experiments with multiple concentrations. The bacteria cultured in concentration of ½ the MIC was then picked to continue the experiments. Secondly, after 35 passages, the ½ the daptomycin MIC of all strains reached a clinically relevant daptomycin concentration of 8 mg/L, and we next used this concentration for the following experiments (Barry et al., 2003; Abhijit et al., 2009). Thirdly, after 60 days of subculturing with increasing sub-inhibitory daptomycin, liquid cultures were streaked onto Colombian agar with 5% sheep blood plates. The most dominant monoclonal colonies were selected and passaged for five generations on antibiotic-free blood agar plates to ensure the stability of DNS strains. Whole-genome sequencing was performed on both the wild-type strains and the DNS strains after five generations of passage on antibiotic-free blood agar plates. The detail steps are described below. The stability of the DNS strain to non-susceptibility to daptomycin was determined by measuring the MIC of daptomycin every 5 passages by passing the DNS strain in antibiotic-free MHB medium for 15 consecutive passages.

### Antimicrobial susceptibility test

*S. aureus* daptomycin MIC were determined by broth microdilution in MHB supplemented with calcium to a final concentration of 50 mg/L. The daptomycin MIC was defined as the lowest concentration that completely inhibited bacterial growth, disregarding tiny buttons of growth per Clinical and Laboratory Standards Institute (CLSI2023) guidelines (Bae et al., 2023). The strains with MIC ≤ 1 mg/L were considered sensitive to daptomycin and non-susceptible if MIC > 1 mg/L (CLSI2023). *S. aureus* ATCC 29213 was used as the reference strain.

### *In vitro* competition experiments

WT strains and paired DNS strains were regulated to the same concentration, 10^6^ colony-forming units per milliliter (CFU/mL) in MHB, take the same amount mixed and incubated at 37 °C for 24 h. At 0 h and 24 h, 100 μL of the mixture was diluted and coated on two MH solid medium (with 0 and 4 mg/L daptomycin, respectively) overnight at 37 °C. The number of colonies on the plates was calculated. To distinguish between the two strains, a resistance assay was conducted using daptomycin-containing plates. The specific method is as follows: Previously confirmed that the WT strains did not grow on daptomycin plates. And in contrast to plates with 4 mg/L daptomycin, double colonies were observed on daptomycin-free plates for cultures collected at 0 hours, indicating that the daptomycin plates inhibited the growth of the WT strains but not the DNS strains. The relative fitness (RF) of the wild type strains and the DNS strains was calculated based on the number of colonies. The RF value calculation formula is: RF=log_10_ (N24/N0)/log_10_ (M24/M0) (Lenski and Travisano, 1994). N and M represent the colonies of DNS strains and WT strains, respectively. 0 and 24 corresponding to 0 h (the competition starting time) and 24 h (after competition), respectively. RF < 1 indicates a fitness cost for the DNS versus WT strain. RF > 1 indicates a fitness advantage of the DNS over the WT strain.

### Growth kinetics experiment

Overnight cultures of WT strains and corresponding DNS pairs (USA300/USA300-DNS, Newman/Newman-DNS, and SA75/SA75-DNS) were grown in Tryptic Soy Broth (TSB; BD, USA) at 37°C with shaking at 220 rpm. Cultures were then resuspended in 10mL of TSB and the optical density (OD_600_) was adjusted to approximately 0.01. The cultures were then incubated in a shaking incubator at 37°C, and aliquots were collected at the following time points: 0, 2, 4, 6, 8, 10, 12, and 24 hours. Each aliquot was serially diluted in 10-fold increments and plated on Columbia agar plates supplemented with 5% sheep blood to quantify bacterial counts. The plates were incubated overnight at 37°C, and the number of colonies was counted to determine the CFU/mL.

### Transmission electron microscopy

Overnight cultures of USA300/USA300-DNS, Newman/Newman-DNS and SA75/SA75-DNS grown in TSB were harvested by centrifugation at 5000 g for 10 minutes, washed with PBS, and fixed in 2.5% glutaraldehyde at 4°C overnight. Following fixation, cells were washed in PBS and post-fixed in 1% osmium tetroxide for 1 hour at 4°C. Fixed cells were processed and embedded in epoxy resin (Epon 812, SPI Supplies, USA) for sectioning. Ultrathin sections (~70 nm) were prepared using an ultramicrotome (Leica EM UC7, Germany) and stained with uranyl acetate and lead citrate. Thin sections were examined by TEM to visualize cell morphology. Cell wall thickness was measured at perpendicular angles along the length of 25 individual cells per strain using ImageJ software.

### Cytochrome C binding assay

Cytochrome C binding assay was carried out as described previously (Vadyvaloo et al., 2004). Cytochrome C and MOPs buffer were purchased from Macklin (Shanghai, China). Briefly, bacterial cells were grown in MHB media for 24h, washed twice with 20 mM MOPs buffer (pH 7.0), and resuspended to OD_600_ 1.0 in MOPs buffer. 500 μL cell suspensions were incubated with 0.5 mg/mL cytochrome C for 10 minutes at room temperature. Reaction mixtures were centrifuged 10,000 g for 5 min and unbound cytochrome C in the supernatants was measured by absorbance at 405 nm.

### Biofilm formation assay

Bacteria were inoculated into 96-well polystyrene plates and incubated at 37°C for 24h to allow biofilm formation. Non-adherent cells were removed by carefully washing each well three times with sterile PBS. Biofilms were fixed with methanol, stained with 0.1% crystal violet for 15 min, and photographed. For quantification, biofilms were solubilized with 30% glacial acetic acid and absorbance at 600 nm was measured. Assays were performed in triplicate wells for each strain. Plate wells inoculated with media alone served as negative controls (Chen et al., 2018).

### Hemolytic activity assay

Cell-free supernatants were collected from 24 h TSB cultures of WT and DNS strains by centrifugation at 12,000 g for 2 minutes. Hemolytic activity was assessed by incubating the supernatants with an equal volume of 2% rabbit red blood cells (RBCs) in PBS for 30 minutes at 37°C. As controls, TSB alone and TSB with 2% Triton X-100 were used to represent 0% and 100% hemolysis, respectively. After incubation, tubes were centrifuged at 4000 g for 10 minutes, and the OD_600_ of the resulting supernatants was measured. Hemolysis was calculated as a percentage of the positive control (Triton X-100).

### *Galleria mellonella* larvae model

*Galleria mellonella* Larvae weighing approximately 250 mg each were used for the infection model (Tianjin Huoyude Bioscience Co., Ltd, Tianjin, China). Groups of 10 larvae were injected with 10 μL of bacterial suspension (1.5×10^8^ CFU/larva) into the last left proleg using a Hamilton syringe. Negative controls included larvae injected with 10 μL of 0.9% (w/v) NaCl. Larvae were monitored for melanization and survival, with death defined as the lack of response to touch. Infected larvae were incubated at 37°C and survival was assessed every 12 h over 3 days. Experiments were repeated at least three times.

### Mice skin abscess model

Five-week-old female BALB/c mice were housed for 1 week before infection to acclimate to the environment. The day before injection, hair was removed from the backs of anesthetized mice. Mice were injected subcutaneously with 10^7^ CFU of WT strains USA300, Newman, and SA75 into the left flank and paired DNS strains USA300-DNS, Newman-DNS, and SA75-DNS into the right flank (n = 5 mice per strain). Control mice received sterile saline injections. Abscess area was measured daily for 5 days using digital calipers. Mice were euthanized at the end of the study. This experiment was independently repeated three times.

### Whole genome sequencing and genomic analysis

Genomic DNA was extracted using the PureLink Genomic DNA Mini Kit (Thermo Fisher Scientific). DNA libraries were prepared with the Nextera DNA Library Preparation Kit (Illumina) and sequenced on an Illumina MiSeq 500-cycle V2 protocol to achieve at least 80x coverage. Raw reads were quality filtered with fastp v0.20.1 (Shifu et al., 2018). Genome assembly of WGS data was performed using Unicycler v0.5.0 (Ryan et al., 2017). Single nucleotide polymorphisms (SNPs) and insertions/deletions were identified relative to the ancestral strain using Snippy v4.6.0 (https://github.com/tseemann/snippy). The Illumina sequences of the six strains are available on NCBI (Accession number: PRJNA1067537).

### Construction of *S. aureus* mutant strains

To construct a single-site mutant of the MprF L341F in the Newman strain, a protocol using the pKOR1 plasmid was followed (Taeok and Olaf, 2005). Briefly, DNA fragments corresponding to the upstream and downstream regions of the MprF L341F site were amplified by PCR, using genomic DNA from Newman-DNS as the template. The PCR products, with added attB1 and attB2 sites at their ends, were purified and ligated to the temperature-sensitive shuttle plasmid pKOR1 using the BP enzyme. The ligated plasmids, containing the MprF L341F site along with its upstream and downstream regions, were extracted from *E. coli* DH5α and electroporated into *S. aureus* RN4220 for modification using Bio-Rad GenePulser electroporator (Cuvette, 1 mm;Voltage, 1.8 kV; capacitance, 25 μF; and resistance, 100 Ω.). Then, the plasmids were transferred into the *S. aureus* strain Newman. The transformants, which underwent allelic replacement of the MprF L341F site, were selected on TSB agar containing 1 mg/L anhydrotetracycline. The mutants were verified through PCR and DNA sequencing (Bae and Schneewind, 2006). The same strategy was used to construct the strain with the site mutation restored (Newman_MprFL341F F341L_) and the single-site mutant of the Cls2 F60S. In addition, the *saeR* deletion mutation in Newman_MprFL341F_ was constructed using a similar method, except that the upstream and downstream fragments were ligated directly without the *saeR* gene fragment. Finally, the complementation of the *saeR* gene was performed using the pLI50 plasmid and digested with KpnI and EcoRI enzymes (Lee et al., 1991).Primers used for the strain construction were listed in **Supplementary Table S2**.

### Daptomycin time-kill assay

Newman_MprFL341F_ and its Δ*saeR* mutant were treated with 4 mg/L (the daptomycin MIC of Newman_MprFL341F_ and its *saeR* knockout strain Newman_MprFL341FΔ_ *_saeR_*) daptomycin in MHB at 37°C with shaking at 220 rpm. Colony counts were determined every 4 h to generate time-kill curves, with Newman strain as control.

### *In vitro* competition assay

To validate the roles of SaeR in conferring competitive fitness, we conducted competition experiments between Newman_MprFL341F_ and Newman_MprFL341FΔ_ *_saeR_* strains in MH liquid media containing 0 mg/L or 2 mg/L daptomycin. Cultures were mixed at a 1:1 ratio and incubated at 37°C with shaking (220 rpm) for 24 h. Since both strains exhibited identical MIC values to daptomycin, we utilized differences in hemolytic phenotype to distinguish between them. The Newman_MprFL341F_ strain displayed more pronounced hemolysis, whereas hemolysis was weaker in the Newman_MprFL341FΔ_ *_saeR_* strain due to *saeR* deletion. To validate strain identity, PCR tests were performed on 10 colonies exhibiting different hemolytic phenotypes. The PCR results correlated with the expected hemolytic phenotypes based on each strain’s genetic differences, validating our approach. Colony counts were used to determine the ratio of surviving strains.

### Statistical analysis

Statistical analyses between the WT and DNS groups were performed using paired t-tests for continuous data, conducted using SPSS software (version X.X, IBM Corp., Chicago, IL, USA). For survival data, Kaplan-Meier survival curves were plotted and compared using the log-rank (Mantel-Cox) test to assess differences in survival rates between the groups. All experiments were repeated at least three times, and results are presented as the mean ± standard deviation (SD), unless otherwise specified. A P-value of less than 0.05 was considered statistically significant. Significance levels were denoted as follows: *P < 0.05, **P < 0.01, ***P < 0.001, ****P < 0.0001.

## Results

### DNS strains exhibit stable non-susceptibility

**Figure 1:**
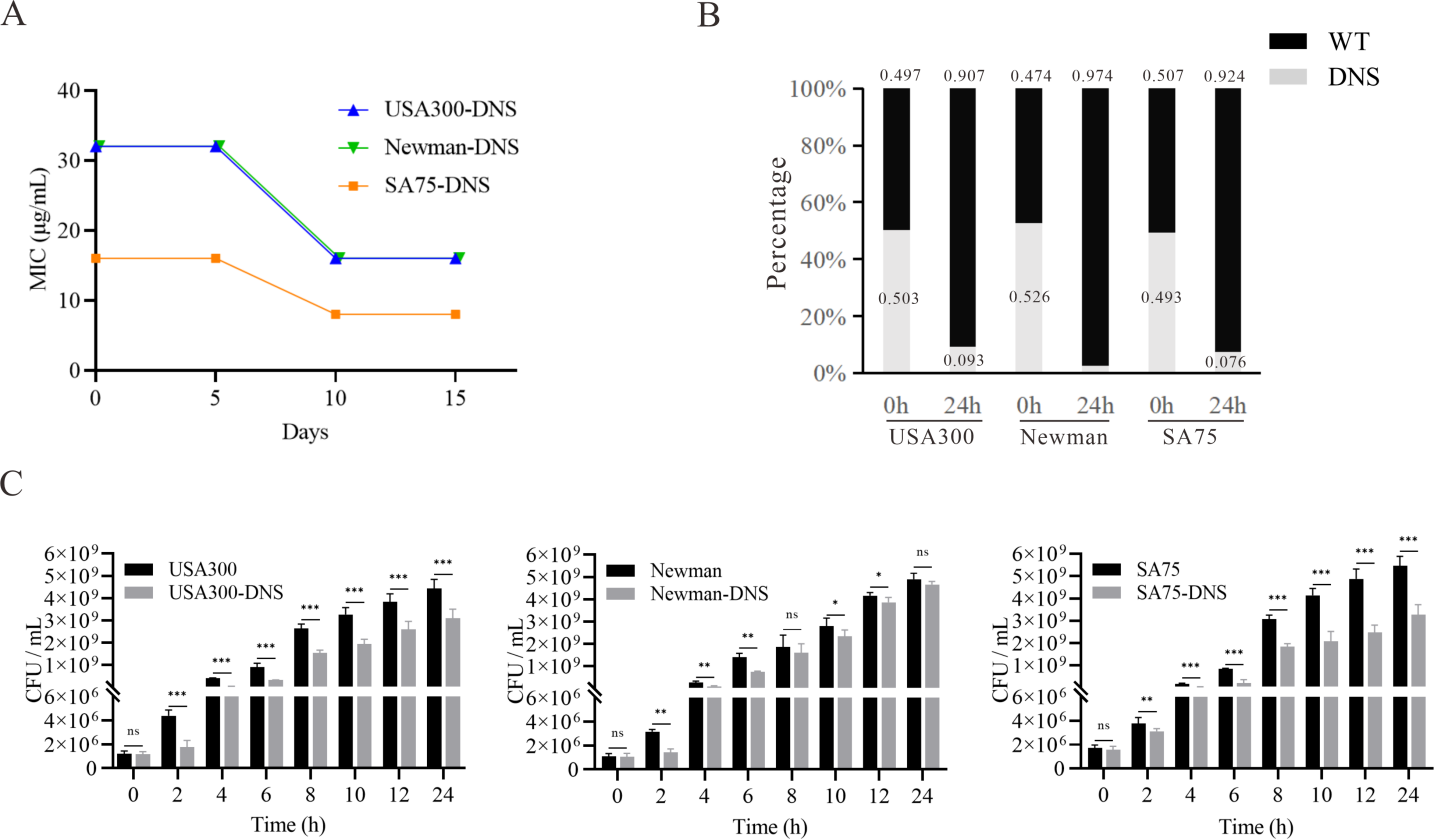
Daptomycin susceptibility and growth characteristics of DNS strains. **(A)** Changes of daptomycin MICs for DNS strains across 5, 10, and 15 consecutive passages in antibiotic-free medium (one passage per day). **(B)** Results of competition experiment between WT and DNS strains at 0 and 24h (start and end of competition). **(C)** Growth dynamics of WT and DNS strains measured by colony-forming units per milliliter (CFU/mL) over a 24-hour period in Tryptic Soy Broth (TSB) at 37°C with shaking. Samples were collected at designated time points (0, 2, 4, 6, 8, 10, 12, 24 hours), serially diluted, and plated on Columbia agar with 5% sheep blood to quantify bacterial counts. These experiments were conducted three times with three replicates per experiment. Unpaired t-tests were performed at each time point to compare growth differences between WT and DNS strains. *P < 0.05, **P < 0.01, ***P < 0.001, ns, not significant.

According to the CLSI criteria, the WT strains USA300 (LAC), Newman, and SA75, with daptomycin MICs of 0.25 mg/L, are classified as susceptible to daptomycin. By sequentially cultivating these WT strains in the presence of progressively increasing daptomycin concentrations, we successfully selected for DNS strains, namely USA300-DNS, Newman-DNS, and SA75-DNS. The MICs changes over time are presented in **Supplementary Figure S1**. And the MICs of the final selected DNS strains against daptomycin were 16 mg/L, 32 mg/L, and 32 mg/L, respectively (**Table 1**). To assess the stability of daptomycin non-susceptibility in these DNS strains, we continued their cultivation in an antibiotic-free medium for 15 consecutive passages. The daptomycin MICs of these DNS strains showed a downward trend over time. Despite this decrease, the strains maintained their non-susceptible status, suggesting that the DNS strains selected by the ALE methodology maintain a certain level of stable non-susceptible to daptomycin (**Figure 1A**).

**Table 1:** Mutations in sites associated with resistance of *S. aureus* to daptomycin.

Strain	Mutations			MIC (mg/L) of daptomycin
	*mprF*	*cls2*	*saeR*	
USA300	NA			0.25
Newman	NA			0.25
SA75	NA			0.25
USA300-DNS	S295L	L52F		16
Newman-DNS	L341F	F60S	N169_Q179del	32
SA75-DNS	L826F	F60L		32

NA, not applicable.

### DNS strains exhibit growth compensation compare to WT strains

We examined the relative competitive growth advantage between WT and DNS strains. Co-incubation of DNS and their corresponding parental WT strains under equivalent inoculum sizes in antibiotic-free Mueller-Hinton medium revealed a significantly lower proportion of DNS strains after 24h (**Figure 1B**). RF values of USA300/USA300-DNS, Newman/Newman-DNS, and SA75/SA75-DNS pairs are 0.73, 0.56, and 0.72, respectively. All RF values were less than 1, implying a fitness cost for DNS strains relative to the WT strains. To gain further insights into the growth dynamics following the development of daptomycin-selected resistance, we measured bacterial growth by tracking CFU/ml under identical inoculation and culture conditions. Instead of relying on optical density (OD_600_), we quantified viable cell counts at various time points. The DNS strains generally exhibited slower growth during the early log phase compared to their respective WT strains (*P* < 0.05), particularly at 2 to 8 hours post-inoculation, as seen in USA300 and Newman strains. However, by the late log phase and stationary phase (12-24 hours), the growth differences became less pronounced, with some DNS strains nearly reaching WT levels (**Figure 1C**). These results suggest that while DNS strains exhibit early growth delays, they may partially compensate for this lag during later growth stages, except for the SA75 strain, which maintained a significant growth deficit compared to its WT counterpart throughout the experiment.

### DNS strains possess thicker cell walls and less negative cell surface charge

**Figure 2:**
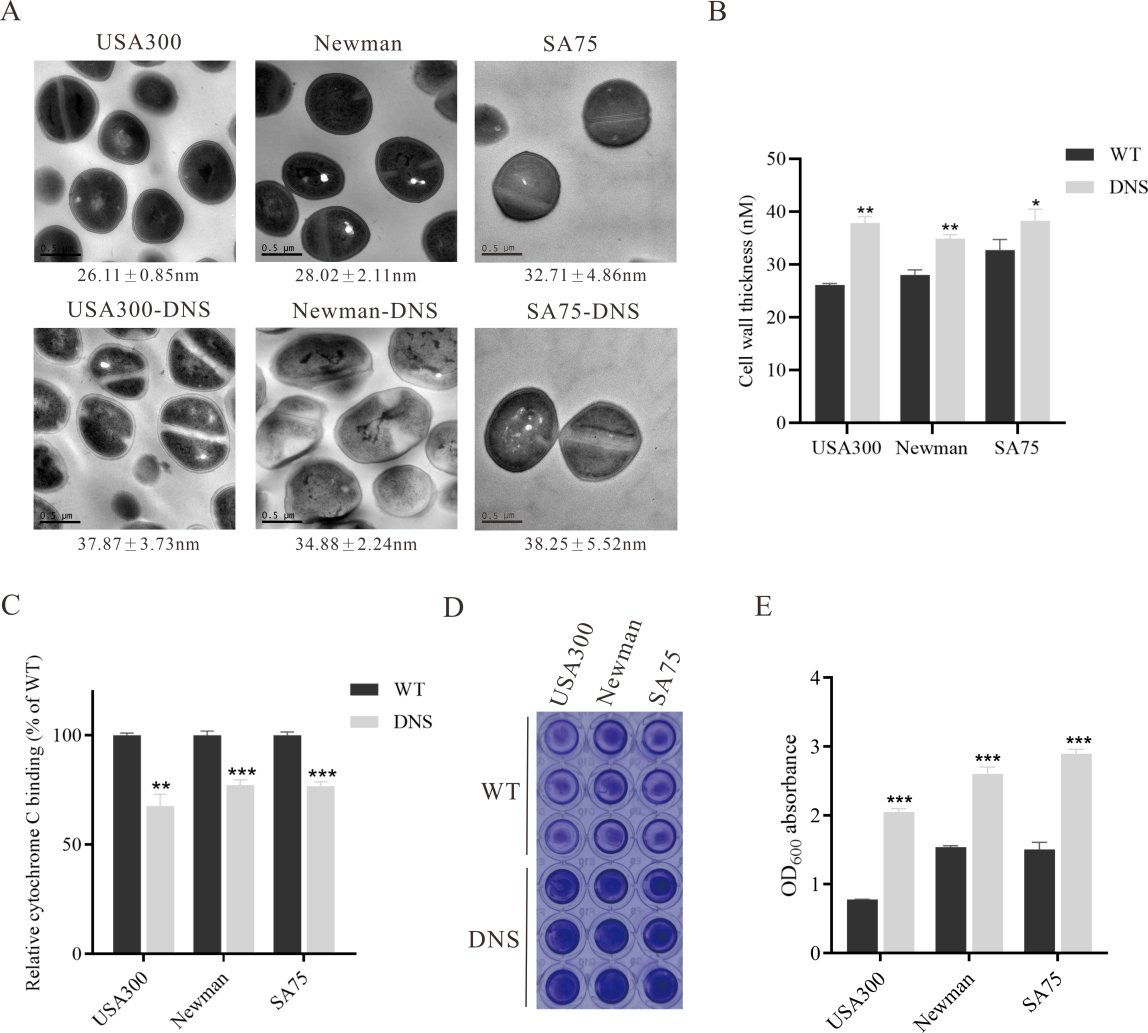
Phenotypic differences between WT and DNS strains. **(A)** Transmission electron microscopy (TEM) images showing the cell wall thickness of WT and DNS strains. Cell wall thickness values are expressed as mean ± standard deviation. Scale bar = 0.5 µm. **(B)** Quantification of cell wall thickness using ImageJ software. DNS strains exhibited significantly increased cell wall thickness compared to WT strains. **(C)** Relative cytochrome C binding to WT and DNS strains (WT binding set to 100%). DNS strains exhibited significantly reduced cytochrome C binding, indicating a decrease in negative surface charge. **(D)** Crystal violet staining of biofilm formation by WT and DNS strains in 96-well plates. **(E)** Quantification of biofilm formation measured by OD_600_ absorbance after solubilization with 30% glacial acetic acid. Data were analyzed using unpaired, two-tailed Student’s t-test. DNS strains demonstrated significantly higher biofilm formation compared to WT strains. *P < 0.05, **P < 0.01, ***P < 0.001.

Thickened cell walls and increased positive cell surface charges are thought to contribute to daptomycin resistance (Yang et al., 2009a; Bertsche et al., 2011). TEM revealed increased cell wall thickness in DNS strains compared to WT strains (**Figure 2A**). Notably, the cell wall of the USA300-DNS strain was 37.87 ± 3.73 nm, significantly thicker than the USA300 WT strain which measured 26.11 ± 0.85 nm (*P* < 0.01) (**Figure 2B**). Similarly, the Newman-DNS and SA75-DNS strains demonstrated increased cell wall thicknesses of 34.88 ± 2.24 nm and 38.25 ± 5.52 nm, respectively, compared to their WT counterparts (Newman WT: 28.02 ± 2.11 nm, SA75 WT: 32.71 ± 4.86 nm; *P* < 0.01 for Newman and *P* < 0.05 for SA75). Cytochrome C can bind to the negative charge on the surface of *S. aureus* cells. An observed decrease in bacterial cytochrome C binding typically indicates an enhanced positive charge on the bacterial surface, which could contribute to resistance against positively charged daptomycin-Ca^2+^ complex (Peschel et al., 1999). The DNS strains exhibited significantly less cytochrome C binding compared to the WT strains, indicating a reduction in the negative charge on their cell surfaces (**Figure 2C**). The USA300-DNS strain showed a 32.34% reduction in cytochrome C binding relative to the WT strain (*P* < 0.01), while the Newman-DNS and SA75-DNS strains displayed approximately 22.79% and 23.22% reductions, respectively (*P* < 0.001 for both). This result signifies a decrease in negative charge on the surface of the DNS strains. Subsequently, we utilized the crystal violet staining method to compare the biofilm formation abilities of WT and DNS strains. As shown in **Figures 2D, E**, DNS strains exhibited a significantly higher biofilm formation capacity than the WT strains (*P* < 0.01).

### Attenuated virulence in DNS strains

**Figure 3:**
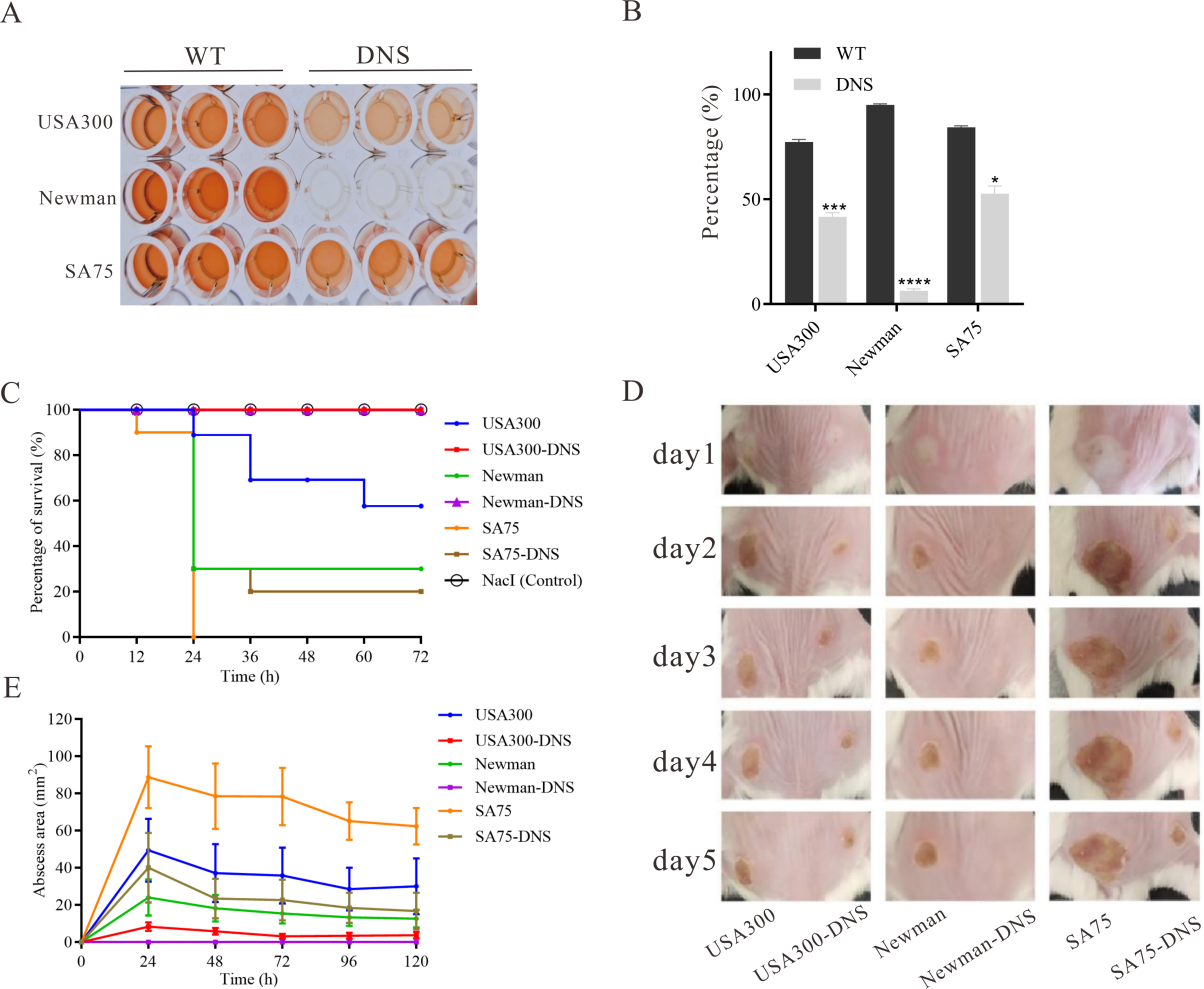
Virulence characteristics of DNS strains. **(A)** Hemolytic activity of WT strains and their corresponding DNS strains. TSB with 2% Triton X-100 served as a positive control, while TSB alone served as the negative control. The experiment was repeated three times with three replicates per repeat. **(B)** Percentage of hemolysis in WT and matching DNS strains. Data were analyzed using unpaired, two-tailed Student’s t-test. *P < 0.05, ***P < 0.001, ****P < 0.0001. **(C)** Survival rates of *Galleria mellonella* larvae following infection with WT and DNS strains were evaluated using Kaplan-Meier survival curves and compared using the log-rank test. **(D)** Representative images of mouse back skin abscesses at the 5 post-inoculation with the strains. **(E)** Skin abscess area in mice caused by WT and DNS strains over a 5-day period.

The hemolytic activity of USA300-DNS and Newman-DNS strains was significantly decreased compared to their respective WT strains (*P* < 0.05), with negligible activity observed for the Newman-DNS strain (**Figures 3A, B**). While the hemolytic activity of the SA75-DNS strain was also reduced, it did not significantly differ from the WT strain. In *Galleria mellonella* larvae model, the 3-day mortality rate of the larvae infected with DNS strains was lower than those infected with WT strains (**Figure 3C**). The mortality rates of larvae infected with DNS strains were significantly lower than those infected with their respective WT counterparts (USA300 vs. USA300-DNS: *P* = 0.0056; Newman vs. Newman-DNS: *P* < 0.0011; SA75 vs. SA75-DNS: *P* = 0.0046). Consistently, in a mice skin abscess model, the abscess area caused by DNS strains was notably smaller than those caused by WT strains (**Figures 3D, E**). Collectively, these results suggest that DNS strains exhibit attenuated virulence compared to WT strains, both *in vitro* and *in vivo*.

### Genetic variants relating to daptomycin resistance in DNS strains

To gain insights into the genetic basis of daptomycin resistance in the DNS strains, we performed whole genome sequencing of WT and DNS strains. Comparative genomics identified multiple single nucleotide polymorphisms (SNPs) in these strains. Specifically, the USA300-DNS strain had missense mutations in the *mprF*, *yfhP*, and *cls2* genes. The Newman-DNS strain had missense or deletion mutations in the *mprF*, *saeR*, *gatC1*, *cls2*, and *yfhP* genes. The SA75-DNS strain had missense or deletion mutations in the *bglK*, *mhqA3*, *mprF*, *bsaA2*, *mutL*, *phaB*, *setC*, *srrA*, *entS*, *yhfP*, *cls2*, *ydjM*, and *purR* genes, as detailed in **Supplementary Table S1**. Among the identified SNPs, we discovered mutations in the *mprF* and *cls2* genes, which have been reported to be associated with daptomycin resistance (**Table 1**). Notably, we also identified a partial deletion in the *saeR* gene in the Newman-DNS strain. The variations were further validated using PCR and Sanger sequencing. These results suggest a potential genetic basis for daptomycin resistance in DNS strains.

### MprF L341F and Cls2 F60S substitutions directly increases daptomycin MIC in Newman strain

Mutations in the *mprF* gene were identified in all three *in vitro* selected DNS strains (**Table 1**). To further investigate the role of the L341F amino acid change in MprF, we introduced this single point mutation into the Newman strain using allelic replacement, yielding Newman_MprFL341F_. This strain showed an increased daptomycin MIC of 4 mg/L, compared to 0.25 mg/L for the parent Newman strain. Reversion to wildtype MprF (Newman_MprFL341FF341L_) restored MIC to 0.25 mg/L. E-test results aligned with broth dilution results (**Table 2**).

**Table 2:** Daptomycin MIC of Newman and its genetic mutated strains tested by broth microdilution and E-test method.

Strains	MIC (mg/L) of daptomycin	
	Broth	E-test
Newman	0.25	0.75
Newman_MprFL341F_	4	6
Newman_MprFL341F_F341L_	0.25	0.75
Newman_MprFL341FΔ_ *_saeR_*	4	6
Newman_Cls2F60S_	2	4
Newman_MprFL341F Cls2F60S_	16	16

Next, we generated Newman_Cls2F60S_ and Newman_MprFL341F Cls2F60S_ strains. Antimicrobial susceptibility testing revealed an increase in the MIC of daptomycin for Newman_Cls2F60S_ and Newman_MprFL341F Cls2F60S_ to 2 µg/ml and 16 µg/ml, respectively (**Table 2**). This suggests that the Cls2 F60S substitution might also be involved in the mechanism of daptomycin resistance. Regrettably, despite several attempts, we were unable to successfully revert the Cls2 F60S substitution.

### *saeR* deletion does not modify MIC or bactericidal effect of daptomycin but enhances competitive growth in presence of daptomycin

**Figure 4:**
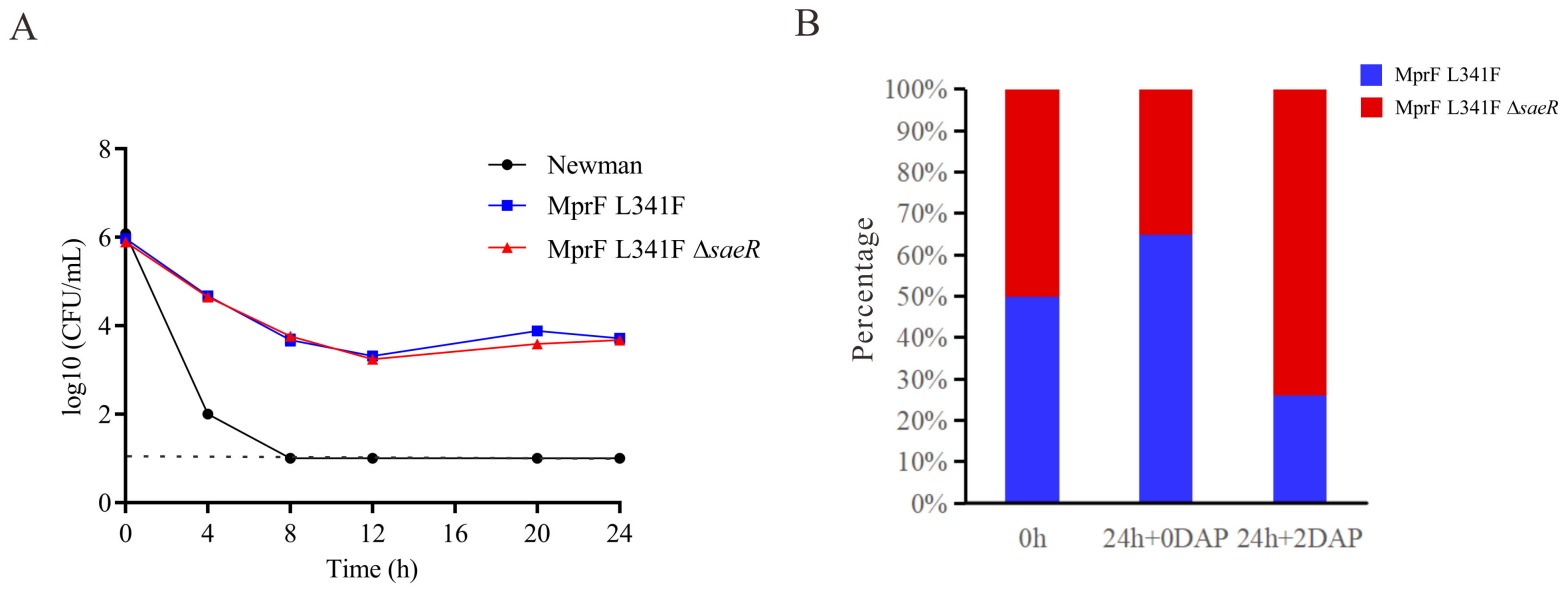
Daptomycin exposure response and competitive growth of Newman with *saeR* Mutation. **(A)** Time–kill analysis showing quantitative bacterial counts during 2 mg/L daptomycin exposure (dashed line indicates detection limit). **(B)** Competition experiment with the proportions of Newman_MprFL341F_ and Newman_MprFL341FΔ_ *_saeR_* strains at 0 h and 24 h in MH broth with or without 2 mg/L daptomycin.

A partial *saeR* deletion was identified in the Newman-DNS strain. To examine the impact of *saeR* on daptomycin resistance, we complemented the *saeR* in the Newman-DNS strain and knocked out the *saeR* in the Newman_MprFL341F_ strain. However, the restoration of *saeR* in Newman-DNS did not modify the daptomycin MIC, which remained at 32 mg/L. Similarly, *saeR* knockout in Newman_MprFL341F_ maintained a daptomycin MIC of 4 mg/L (**Table 2**). These results suggest that the *saeR* mutation does not impact the daptomycin MIC in Newman. Despite the lack of impact on daptomycin MIC, we further examined the effect of *saeR* on bactericidal activity of daptomycin. As shown in **Figure 4A**, no growth was observed for Newman after 8 h exposure to daptomycin concentration of 4 mg/L. Newman_MprFL341F_ and its *saeR*-negative mutant strain (Newman_MprFL341FΔ_ *_saeR_*), showed similar colony counts at each timepoint, indicating that *saeR* deletion does not influence the bactericidal effect of daptomycin.

However, competitive growth assays with Newman_MprFL341F_ and its *saeR* knockout strains in broth with or without 2 mg/L daptomycin (**Figure 4B**) yielded different results. Relative fitness values were 0.91 without daptomycin and 1.18 with 2 mg/L daptomycin. This suggests Newman_MprFL341FΔ_ *_saeR_* exhibits inferior growth versus Newman_MprFL341F_ without daptomycin but outcompetes it in the presence of daptomycin. In conclusion, while *saeR* deletion does not alter daptomycin susceptibility, it enhances the competitive growth of *S. aureus* in the presence of daptomycin.

## Discussion

Daptomycin is one of the first-line drugs used for MRSA treatment, but emergence of daptomycin non-susceptible MRSA strains can directly lead to treatment failure (Skiest, 2006). Understanding the phenotypic characteristics and resistance mechanisms of daptomycin non-susceptible strains is crucial for clinical management and delaying further drug resistance. The ALE strategy rapidly generates non-susceptible strains (Mishra et al., 2012). In this study, we selected three DNS strains *in vitro* and demonstrated stability of daptomycin resistance. Notably, there was a 2-fold decrease in MIC when cultured without daptomycin (**Figure 1A**), suggesting an adaptive resistance mechanism or possibly a reversion to the wild-type allele. In this study, we found that these DNS strains had mutations in the *mprF* gene. Previous *in vitro* studies determined that dual *mprF* mutations (like S295 L+L826F) in daptomycin non-susceptible strains reversed these strains susceptible to daptomycin, especially in the case of β-lactam pressure (Yang et al., 2018; Mishra et al., 2021). In this study, we have performed sequencing of the *mprF* gene for the DNS strains after passage, but found no secondary mutations. Probably because the DNS strains remained higher level of non-susceptible to daptomycin after 15 generations due to no pressure was applied.

Bacteria often acquire drug resistance via gene mutation or horizontal transfer, accompanied by fitness costs like slower growth or reduced virulence (Guo et al., 2012). In our study, we observed a delayed growth and competitive disadvantage without antimicrobials in DNS versus WT strains. When daptomycin pressure is removed, susceptible strains outcompete DNS strains due to growth advantages. This feature was also observed for a DNS strain derived from a clinical blood culture by Melanie Roch et al., which they attributed for clinical reduced dissemination of daptomycin resistance as well as the absence of daptomycin reported outbreaks (Roch et al., 2017). Additionally, *in vitro* and *in vivo* pathogenicity were both weakened in DNS strains. These results suggest that *S. aureus* compensates for losses in growth and virulence acquisition upon developing daptomycin non-susceptibility, consistent with other reports (Cameron et al., 2015; Li et al., 2017; Roch et al., 2017).

Our study revealed that DNS strains exhibited cell wall thickening and an increase in positive cell surface charge, these characteristic consistent with previously clinically isolated DNS strains (Bayer et al., 2013). Additionally, we observed a significant thickening of biofilm formation in the three DNS strains. This enhanced biofilm formation ability could contribute to the persistence and resistance of DNS strains (Das et al., 2010). Increased biofilm formation suggests that bacteria are more likely to adhere to the surfaces of infected tissues and various implanted devices, leading to the chronic or recurrent infections (Percival et al., 2015).

Analysis of whole-genome sequencing data in our study uncovered several substitutions in daptomycin resistance-related genes in DNS strains. These included MprF S295L, MprF L341F, MprF L826F, Cls2 L52F, Cls2 F60S, and Cls2 F60L substitutions. Previous studies have demonstrated a strong link between the *mprF* gene and daptomycin resistance (Yang et al., 2009b; Rubio et al., 2011). Overexpression of *mprF* leads to diminished sensitivity to daptomycin, and conversely, knockout of *mprF* expression results in enhanced sensitivity. However, not all mutation sites in the *mprF* gene confer daptomycin resistance. Only P314L, S337L, T345A, and V351E substitutions have been confirmed to cause daptomycin resistance in *S. aureus* SA113 (Ernst et al., 2018). The roles of MprF S295L and MprF L826F substitutions in bacterial daptomycin resistance have been reported previously (Yang et al., 2013; Yang et al., 2018). Although there have been found in clinical isolates, the significance of MprF L341F, Cls2 F60S, and Cls2 F60L substitutions is not well understood and remains to be elucidated. Indeed, mutations at the 341 locus are less frequently found in daptomycin-induced *S. aureus* strains, most of the mutations were S295 L, P314 L, S337 L, T345A, V351E, I420 N, L826 F (Ernst et al., 2018; Erica et al., 2019; Vladimir et al., 2023). One study identified two *S. aureus* with daptomycin MICs>2 mg/L (only one amino acid substitution in MprF at position 341, L341S), which were isolated from patients who had been treated with daptomycin and failed therapy (Bayer et al., 2014). Another clinical isolate of *S. aureus* containing the L341S mutation also showed a MIC of 4 mg/L for daptomycin (Nagendra et al., 2011). These studies suggest that mutations in this amino acid will most likely cause higher level of daptomycin non-susceptibility. To demonstrate this, we performed *in situ* mutation of this site in Newman. Our results showed that the MprF L341F substitution increased the daptomycin MIC of Newman from 0.25 mg/L to 4 mg/L. To eliminate the possibility of daptomycin resistance caused by other substitutions in the genome during construction, we reverted the L341F site of the Newman_MprFL341F_ strain back to its original state, thereby creating the strain Newman_MprFL341F F341L_. The MIC value of daptomycin for this reverted strain returned to the original 0.25 mg/L. Our results may explain the emergence of this phenomenon, that the MprF L341 site mutation can directly impacts bacterial sensitivity to daptomycin. The amino acid at position 341 does not involve the flippase and synthetase domain of MprF protein. One study indicated that amino acid mutations located at the domain junction do not change the level and translocation of L-PG, but may increase the type of flippase substrate in MprF, such as alanine-PG (Ernst et al., 2018). Whether the amino acid mutation at position 341 also has such a role needs to be further investigated.

Mutations in the *cls2* gene have been identified in several daptomycin-resistant bacteria. The K61 deletion of Cls2 and the R218Q mutation located in the D domain of phospholipase are involved in *S. aureus* daptomycin resistance (Peleg et al., 2012). In *S. aureus*, other substitutions such as T33N, L52F, A23V, and F60S have also been detected. Research has indicated that T33N and L52F substitutions in Cls2 can elevate the daptomycin MIC value in *S. aureus*. Daptomycin treated experiments have shown that, at a particular concentration of daptomycin, the Cls2 A23V mutant strain may initially be killed in part by daptomycin, but surviving bacteria can resume reproduction over time (Jiang et al., 2019). We also identified substitutions at the Cls2 F60S locus in our study. Although a single mutant strain at this locus was not successfully constructed in previous studies, we were able to construct one based on the Newman WT strain in our study. This single mutant strain exhibited a daptomycin MIC of 2 mg/L. Furthermore, the Newman_MprF L341F Cls2 F60S_ double mutant strain exhibited a daptomycin MIC of 16 mg/L, identical to the post-passage Newman-DNS strain. However, as we have not constructed restore strains at this locus, it is not yet clear whether other substitutions may have contributed to daptomycin resistance. The amino acid at position 60 is located in the transmembrane domain of the Cls2 protein. It has been proved that Cls2 amino acid substitutions led to increased cardiolipin synthase activity and increased cardiolipin production that was subsequently causing a reduction in PG (Jiang et al., 2019).

We detected a partial frame deletion in the *saeR* gene of the Newman-DNS strain. This observation could explain the noted low virulence of the Newman-DNS strain. Other research has also reported that the *saeR* gene mutates following the *mprF* gene in DNS strains, suggesting this is not a coincidental occurrence (Miller et al., 2021). Therefore, we assessed the role of *saeR* in bacterial susceptibility to daptomycin. However, neither knockout nor overexpression *saeR* altered the bacterial daptomycin MIC. Interestingly, in a competitive growth assay in MH liquid medium containing 2 mg/L of daptomycin, the strain with the *saeR* gene deletion demonstrated a competitive growth advantage. This suggests that mutations in the *saeR* gene may serve as a compensatory mechanism for bacteria to adapt to daptomycin.

In fact, earlier studies have found that the additional point mutations in other loci emerged shortly after the emergence of mutations in *mprF*, which further decreased daptomycin susceptibility (Friedman et al., 2006). Double mutations in the *mprF* and *cls 2* genes are particularly common in some strains with daptomycin-induced resistance, this phenomenon also reflected by our study (Erica et al., 2019). Our study demonstrated that the mutations in the secondary *cls 2* gene can further decreased daptomycin susceptibility, while the *saeR* gene could not.

In summary, all the phenotypic results above demonstrated that DNS strains selected by the ALE strategy exhibits similar phenotypes to clinically isolated DNS strains. The MprF L341F substitution directly enhances daptomycin MIC of Newman, while deletion of *saeR* does not change bacterial sensitivity to daptomycin but enhances the competitive growth capacity of bacteria in the presence of daptomycin. These findings help to shed light on the dynamics of bacterial adaptation to daptomycin, as well as the role of specific genetic changes in this process.

## Data Availability

The datasets presented in this study can be found in online repositories. The names of the repository/repositories and accession number(s) can be found in the article/**Supplementary Material**.
