# Supplementary material for: Phenotypic and genetic characterization of daptomycin non-susceptible Staphylococcus aureus strains selected by adaptive laboratory evolution
Source: Front Cell Infect Microbiol. 2024 Oct 24;14:1453233. doi: 10.3389/fcimb.2024.1453233 (PMC11540788; doi:10.3389/fcimb.2024.1453233)
Supplement: Supplementary file 1 [file DataSheet1.pdf]

Supplementary Materials

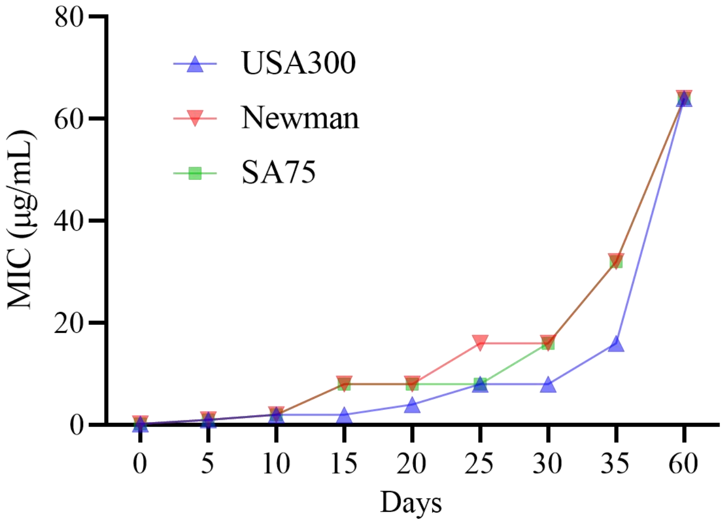

Figure S1. Progression of minimum inhibitory concentration (MIC) of daptomycin over time for USA300, Newman, and SA75 strains. MIC was monitored regularly throughout the 60-day experiment.

Table S1. Daptomycin related non-synonymous mutational differences between WT and DNS strain pairs identified by whole genome sequencing.

Strain	POS	TYPE	REF	ALT	GENE	PRODUCT	EFFECT
USA300-DNS	60873	snp	G	A	*mprF*	Phosphatidylglycerol lysyltransferase	missense_variant Ser295Leu
USA300-DNS	108537	snp	C	T	*yfhP*	putative protein YfhP	missense_variant Gly43Arg
USA300-DNS	27562	snp	A	C	*Cls_2*	Cardiolipin synthase	missense_variant Leu52Phe
Newman-DNS	260444	snp	A	T	*mprF*	Phosphatidylglycerol lysyltransferase	missense_variant Leu341Phe
Newman-DNS	205642	del	TTTTTCAAGTAATTCTGATTTAGAAATAACTTCA	T	*saeR*	Response regulator SaeR	disruptive_inframe_deletion 507_539delTGAAGTTATTTCTAAATCAGAATTACTTGAAAA p.Asn169_Glu179del
Newman-DNS	206191	snp	T	C		hypothetical protein	missense_variant Lys147Glu
Newman-DNS	120045	snp	G	A	*gatC_1*	PTS system galactitol-specific EIIC component	missense_variant Met70Ile
Newman-DNS	25901	snp	T	G		hypothetical protein	missense_variant Cys18Gly
Newman-DNS	25908	snp	A	G		hypothetical protein	missense_variant Asp20Gly
Newman-DNS	25971	snp	T	G		hypothetical protein	missense_variant Val41Gly
Newman-DNS	50134	snp	T	C		hypothetical protein	missense_variant Leu499Ser
Newman-DNS	27551	snp	T	C	*cls_2*	Cardiolipin synthase	missense_variant Phe60Ser
Newman-DNS	95238	snp	T	C		hypothetical protein	missense_variant Ile125Thr
Newman-DNS	7092	snp	T	C	*yfhP*	putative protein YfhP	missense_variant Asp44Gly
Newman-DNS	347	snp	C	T		hypothetical protein	missense_variant Ser155Asn
SA75-DNS	141812	ins	A	AC	*bglK*	Beta-glucoside kinase	frameshift_variaAla139fs
SA75-DNS	109081	snp	G	A	*mhqA_3*	Putative ring-cleaving	missense_variant Met209Ile

						dioxygenase MhqA	
SA75-DNS	158891	snp	G	A	*mprF*	Phosphatidylglycerol lysyltransferase	missense_variant Leu826Phe
SA75-DNS	224910	del	CA	C	*bsaA_2*	Glutathione peroxidase BsaA	frameshift_variaThr147fs
SA75-DNS	233469	snp	T	A	*mutL*	DNA mismatch repair protein MutL	stop_gained Lys428*
SA75-DNS	251337	snp	T	C	*phaB*	Acetoacetyl-CoA reductase	splice_region_variant&stop_retained_variant Ter235Ter
SA75-DNS	440904	del	CA	C		hypothetical protein	frameshift_variant Lys5fs
SA75-DNS	174647	del	CA	C	*setC*	Sugar efflux transporter C	frameshift_variant c.1192delA p.Ile398fs
SA75-DNS	240118	snp	A	G		hypothetical protein	missense_variantSer88Pro
SA75-DNS	2734	snp	T	C	*srrA*	Transcriptional regulatory protein SrrA	missense_variant Asp53Gly
SA75-DNS	18747	del	TA	T	*entS*	Enterobactin exporter EntS	frameshift_variant Leu366fs
SA75-DNS	68090	snp	A	T		IS1182 family transposase ISSau3	missense_variant Cys39Ser
SA75-DNS	13114	snp	G	A	*yhfP*	Putative quinone oxidoreductase YhfP	missense_variant Gly227Asp
SA75-DNS	80265	snp	G	T	*cls_2*	Cardiolipin synthase	missense_variant Phe60Leu
SA75-DNS	7819	del	CA	C	*ydjM*	Inner membrane protein YdjM	frameshift_variant Phe61fs
SA75-DNS	20592	snp	T	C		hypothetical protein	missense_variantAsp11Gly
SA75-DNS	20816	del	AT	A		hypothetical protein	frameshift_variant Asn119fs
SA75-DNS	13734	ins	C	CT	*purR*	Pur operon repressor	frameshift_variant Val30fs

Table S2. Primers used for the strain construction

Primer	Primer sequences
saeR-UF[a]	GGGGACAAGTTTGTACAAAAAAGCAGGCT<br>TGAGAAGGATACCCATAA
saeR-UR[a]	ttactgatcgtggatgatgaCCTATGCGTATTAAGGAA
saeR-DF[a]	ttccttaatacgcataggTCATCATCCACGATCAGTAA
saeR-DR[a]	GGGGACCACTTTGTACAAGAAAGCTGGGT<br>TAGCTTTAGGTGCTTGTGGT
saeR-C-F[b]	gtcttcaagaattcgagctcggtacc<br>CCAGTCATCGCTAACAATAC
saeR-C-R[b]	ctgcaggtcgactctagaggatcc<br>TAACACCATTATCGGCTCCT
mprF-341-F[c]	GGGGACAAGTTTGTACAAAAAAGCAGGCT<br>TCACATTGTATCGGGAGTTA
mprF-341-R[c]	GGGGACCACTTTGTACAAGAAAGCTGGGT<br>TCGACTGAAATGGCATCA
cls2-60-F[c]	GGGGACAAGTTTGTACAAAAAAGCAGGCT<br>CATTTATTAGTTATGGTGGTAGT
cls2-60-R[c]	GGGGACCACTTTGTACAAGAAAGCTGGGT<br>ATCAGGTATGAAATAGGGAGA

[a] primers used for gene deletion

[b] primers used for gene complementation

[c] primers used for gene single site mutation
